# Reply to comments on Monitoring vaccination coverage: Defining the role of surveys

**DOI:** 10.1016/j.vaccine.2016.09.067

**Published:** 2016-12-07

**Authors:** Felicity T. Cutts, Pierre Claquin, M. Carolina Danovaro-Holliday, Dale A. Rhoda

**Affiliations:** London School of Hygiene and Tropical Medicine, UK; Frontline Field Epidemiology Training Programme, Tephinet/CDC, Bangladesh; World Health Organization, Geneva, Switzerland; Biostat Global Consulting, OH, USA

Dear Editor,

We thank Pond and Mounier-Jack for their comments on our paper, “Monitoring vaccination coverage: Defining the role of surveys” [1]. We agree that for many countries, administrative estimates of coverage are greatly inflated and misleading for programme planning purposes. The robustness of the WHO-UNICEF estimates of national immunization coverage (WUENIC) depends on the quality of the underlying data reviewed, which include administrative reports, as well as probability and non-probability sample surveys. In 2012, the Grade of Confidence (GoC) was introduced as a means of conveying uncertainty in WUENIC [2] and is low in the seven conflict-affected countries listed by Pond and Mounier-Jack. Table 1 shows that in five of these countries, vaccination cards were available for less than half the children surveyed; when card availability is low, it is particularly difficult to compare coverage trends. For example, in Nigeria, the proportion of children with DTP3 according to card was similar in surveys in 2010, 2011 and 2013, but in the EPI survey of 2010 a verbal history of vaccination was reported for 43% of children, more than double that of previous or subsequent surveys. Elsewhere, results from surveys did not always match expected trends (e.g. no apparent fall in coverage between surveys despite a 7 month stockout of DTP in one country), and some results were very unlikely (e.g. zero dropout between DTP1 and DTP3 in one Multiple Indicator Cluster Survey (MICS) (data from country reports at http://apps.who.int/immunization_monitoring/globalsummary/wucoveragecountrylist.html)).

The updated WHO guidelines on vaccination coverage surveys (http://www.who.int/immunization/monitoring_surveillance/Vaccination_coverage_cluster_survey_with_annexes.pdf) discuss the challenges of using a new survey to compare with an older one, particularly an immunization coverage survey – these often lacked information on likely biases and confidence intervals were either not reported or not very meaningful from non-probability samples. The best way to compare results from different surveys is to plan a pair of surveys for such a purpose and work very hard to ensure standardised, well-documented and high quality data collection in both. Pond and Mounier-Jack suggest that two such surveys are feasible within each 5 years period. We would be reluctant to stipulate any particular interval as the usefulness of repeat surveys will depend in part on the likelihood of a change in coverage having occurred (which can be predicted from monitoring other indicators) [1] and the availability of accurate documentation of vaccination status on home-based or clinic records. Most of all, surveys should lead to action to strengthen programme performance and this is likely the weakest link in many countries, including those affected by conflict.

We also question whether frequent conduct of high-quality surveys is always the best investment, particularly when countries may not use results to improve EPI performance. In the Americas, strong progress towards programme goals has been attributed to technical oversight, partnership and coordination to strengthen routine information systems and the continuous monitoring of administrative data (including numerators separate from denominators), surveillance and public health laboratory networks, as well as pooled vaccine purchase [3], [4]. The Pan American Health Organization (PAHO) rarely recommended or funded surveys [4].

We encourage the global community to continue its support to improve monitoring systems as well as surveys, while building-up the evidence regarding the best uses of vaccination coverage surveys and other monitoring tools, without losing focus on the actual implementation of strategies proven to improve immunization programme performance.

## Conflict of interest statement

The authors declare that they have no conflict of interest.

## Figures and Tables

**Table 1:** Surveys reviewed for WUENIC in 7 countries, 2008–2015, children aged 12–23 months.

Country	Year of WUENIC	Year of Survey	Survey type	% cards	DTP3% by card	DTP3% History	DTP3% Total
Afghanistan	2012	2013	EPI	66	54	6	60
	2010	2010–11	MICS	31	32	9	41

Cote d’Ivoire	2014	2015	EPI review	91	70	6	76
	2013	2014	Post-SIA	75	61	11	82
	2012	2013	EPI	88	78	4	82
	2011	2011–12	DHS	74	56	8	64

Central African	2011	2012	EPI	50	41	6	47
Republic	2009	2010	MICS	32	16	16	32

Democratic	2012	2013–14	DHS	26	24	36	60
Republic of Congo	2011	2012	EPI	35	21	56	77
	2009	2010	MICS	43	37	25	62

Mali	2011	2012–13	DHS	38	29	34	63
	2009	2010	MICS	59	49	23	72
	2008	2009–10	EPI	65	47	28	75

Nigeria	2012	2013	DHS	28	22	16	38
	2010	2011	MICS	24	26	18	45
	2009	2010	EPI	40	25	43	68
	2007	2008	DHS	26	20	15	35

Pakistan	2013	2014–15	PSLM	n/a	65	23	88
	2012	2012–13	DHS	36	32	33	65
	2012	2013–14	PSLM	n/a	61	20	81
	2010	2010–11	PSLM	n/a	56	19	85
	2007	2008–9	PSLM	n/a	51	33	84

WUENIC: WHO/UNICEF Estimates of National Immunization Coverage.

DTP3: third dose of diphtheria-tetanus-pertussis vaccine (results are for children aged 12–23 months).

EPI: Expanded Programme on Immunization.

MICS: UNICEF Multiple Indicator Cluster Survey.

DHS: Demographic and Health Survey.

PSLM: Pakistan Social and Living Standards Measurement Survey.

## References

[1] Cutts F.T., Claquin P., Danovaro-Holliday M.C., Rhoda D.A. (2016). Monitoring vaccination coverage: defining the role of surveys. Vaccine.

[2] Brown D.W., Burton A., Gacic-Dobo M., Karimov R.I. (2013). An introduction to the grade of confidence used to characterize uncertainty around the WHO and UNICEF estimates of national immunization coverage. Open Public Health J.

[3] Dietz V., Venczel L., Izurieta H., Stroh G., Zell E.R., Monterroso E. (2004). Assessing and monitoring vaccination coverage levels: lessons from the Americas. Rev Panam Salud Publica.

[4] Tambini G., Andrus J.K., Fitzsimmons J.W., Roses Periago M. (2006). Regional immunization programs as a model for strengthening cooperation among nations. Rev Panam Salud Publica.

